# Knowledge, Attitudes and Practice of Desalinated Water among Professionals in Health and Water Departments in Shengsi, China: A Qualitative Study

**DOI:** 10.1371/journal.pone.0118360

**Published:** 2015-04-13

**Authors:** Tao Chen, Qiqi Wang, Yu Qin, Xi Chen, Xiaoxiong Yang, Wei Lou, Mikang Zhou, Guangxue He, Kai Lu

**Affiliations:** 1 National Institute for Viral Disease Control and Prevention, Chinese Center for Disease Control and Prevention, Beijing, China; 2 Chinese Center for Disease Control and Prevention, Beijing, China; 3 Institute for Environmental Health and Related Product Safety, Chinese Center for Disease Control and Prevention, Beijing, China; 4 Shengsi County Center for Disease Control and Prevention, Zhejiang, China; The Ohio State University, UNITED STATES

## Abstract

Desalination has been considered as an essential way to solve water stress all over the world. Most of previous studies focused on its environmental impacts, energy consumption and desalination technologies rather than human health. However, the safety of desalinated water remains unclear. This study was undertaken to investigate the knowledge, attitude and practice (KAP) of the residents in an island county in eastern China to desalinated water. Seventeen people working in medical and water industries were recruited, and focus group discussion and in-depth interview were conducted among them. Our results showed that the majority of people interviewed knew the definition and local supply pattern of desalinated water, while some of them showed some concern about the safety and nutrition of desalinated water. Current drinking water standard has no specific item for desalination, so we strongly suggest issuing a standard for desalinated water.

## Introduction

The insufficiency of freshwater has become a growing concern all over the world. Presently, over one-third of the world’s population lives in water-stressed countries and by 2025, this figure is predicted to rise to nearly two thirds [1]. Desalination, as an open source incremental technology, has been considered as an essential way to solve the global water crisis. It is estimated that by 2016, the global water production by desalination will exceed 38 billion cubic meters per year, nearly twice the rate of global water production in 2008[2]. The desalination industry has begun to take shape in China in recent decades. During the 11th Five-Year Plan period (2006–2010), the average annual growth of desalination capacity has risen to 60%. More than 70 seawater desalination plants with projected desalination capacity of 600,000 m^3^ per day had been built by the end of 2010. According to the 12th Five-Year (2010–2015) Plan for the scientific and technological development of seawater desalinization [3], an innovative system would be established during the period, and desalinization technology should reach an advanced level. It is estimated in the plan that the desalinization industry in China will produce a market value between $70 billion and $95 billion at the end of 2015. In the next 20 years, China will remain one of the fastest growing markets in the world. Together with this plan, the State Council General Office, the National Development and Reform Commission and other departments, have successively promulgated several proposals and plans since 2012, highlighting the country's determination to develop the seawater desalination industry.

China has a long coastline with many islands. About 40% of the total population lives in coastal area, therefore cities and islands who are suffering from the fresh water shortage have acted to develop desalination projects. Desalination, taking the salt out of seawater to make it drinkable, can augment and replenish the amount of freshwater and guarantee consistent supply of drinking and industrial water in coastal areas without time and climate limit.

In 1997, a seawater reverse osmosis (SWRO) desalination plant with 500 tons output per day was constructed in Shengsi County, Zhejiang province. After this, more SWRO desalination plants were built in other islands in Shengsi County [4]. Desalinated water has become one of the main water sources there [5]. The knowledge, attitude and practice (KAP) of local people to desalinated water will affect the compliance and accessibility in promoting this new source of water. In our study, focus group discussion and in-depth interview were conducted to understand the related KAP of local professionals who were working in health and water departments to guide the promotion of desalinated water use, lay a foundation of further studies and find a basis for quantitative questionnaire survey in the future.

## Data and Methods

### Ethics Statement

All study procedures were reviewed and approved by the Ethical Review Committee of Institute for Environmental Health and Related Product Safety, Chinese Center for Disease Control and Prevention. During the approval process for this study, the Ethical Review Committee was explicit about the need to conduct an anonymous survey. Because the major risk associated with this study was privacy and possible anxiety associated with signing of forms, we received approval from the Ethical Review Committee to use verbal consent procedures. All participants gave their verbal informed consent to be interviewed in the presence of a local staff from center for disease control and prevention (CDC) and the interview staff. The interviewer signed the consent form stating that the participant had understood the purpose and other details of the study and had voluntarily agreed to be interviewed.

### Study sites and area

**Fig 1:**
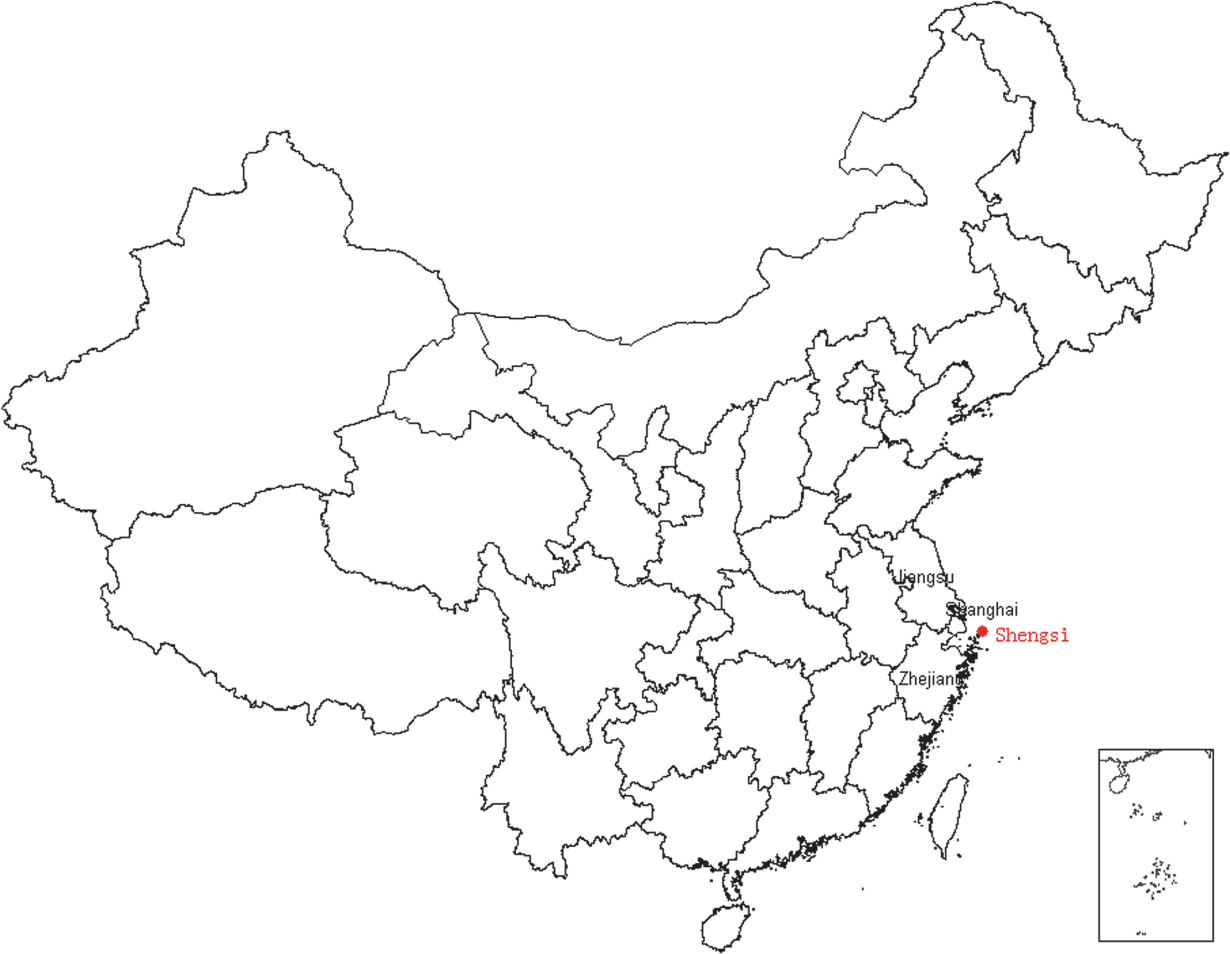
The Location of Shengsi. The red spot in this figure is Shengsi County.

Shengsi County, where the study field set, is located in east of China (Fig 1). It is composed by 404 islands and covers a total area of 8824 square kilometers, and the land accounts for 88 square kilometers. However, Shengsi County is facing a severe shortage of the resource of terrestrial water. With about 1000 millimeters annual rainfall, it has become one of the water-stressed island counties in China.

### Participants and Data collection

The subjects from this study were enrolled in local administrative department, water conservancy department, waterplants, CDC, hospitals and maternity and child care centers according to a purposive sampling method. They were better educated, with better income, more knowledgeable about desalination and health, so their concerns and misunderstanding of desalinated water was supposed to be the cardinal and core problems in promoting desalinated water at present. Owing to their position, they were respected and were trendsetters in using desalinated water. Meanwhile, due to the content of their work, medical staffs were more sensitive to notice the health risks of desalinated water and easier knowing the concerns of ordinary people. People were eligible for enrolment if they (i) lived in Shengsi for more than 20 years, (ii) had capacity to express and communicate clearly. 17 participants were recruited after informed consent. Twelve medical staff who were from county CDC, hospitals and maternity and child care centers were involved in focus group discussion (FGD). Taking account of the impossibility of trauma caused by drinking desalinated water, while all the medical staffs from maternity and child care center are major in gynecology and obstetrics, we only recruited subjects from department of internal medicine and physical examination in Shengsi hospital. In order to ensure the homogeneity of study, respondents from the same institution were divided into one group. No obvious disagreement was found during the course of collecting data; so no more groups were added. A total of 3 focus group discussions were completed and each FGD lasted for 0.5 to 1.5 hours. The in-depth interview was conducted among the directors of health administrative department, water conservancy department and waterplants. In total 5 directors were interviewed for 30 to 50 minutes. Besides the knowledge, attitude and practice of desalinated water, the policy, measures, social repercussions and the next steps in promoting this new source of water were also asked during the in-depth interview.

### Analysis

Original data were recorded and verified after each interview by four researchers. Thematic framework analysis method was used with MAXqda software. Transcripts were analyzed independently by these four researchers through line-by-line coding. A series of common themes were determined (Table 1), and saturation was reached when no new themes were identified. Then researchers classified and finally analyzed, discussed the results and illustrated them in the original words of the people interviewed.

**Table 1:** Summary of themes.

**(1) Knowledge about desalinated water**
Definition of desalinated water
Water Supply Information
Safety of desalinated water
**(2) Attitudes to desalinated water**
Attitude to safety
Attitude towards nutrition
Attitude to government
**(3) Practice of desalinated water usage**

### Reliability of result

The interview protocol was discussed and revised before formal study and a pre-survey was conducted to test its feasibility. The investigators received training before the implementation of the study. Although a larger sample would be needed to get more comprehensive results, substantive topics and great similarity of these topics were evident after interviews with 17 participants, thus indicating a sufficient level of saturation.

## Results

Socio-demographic information is presented in Table 2.

**Table 2:** Participant characteristics (N = 17).

**Characteristics**		**n(%)**
**Gender**	Male	13(76)
	Female	4(24)
**Organization**	Water Industry	3(18)
	Health Industry	14(82)
**Post**	Manager/Chief	12(70)
	Staff	5(30)
**Location**	Shengsi	17(100)

### Knowledge about desalinated water

#### Definition of desalinated water

Seawater desalination is a process to remove its excess salt and minerals to get fresh water. The majority of respondents knew the definition of desalinated water. One of them said, "*That is to take away the salt by some technique*.” However, no one knew the technology used in desalinated water production except 3 people with related work in waterplants in Shengsi, in which reverse osmosis membrane technique was used.

#### Water Supply Information

The amount of supplied desalinated water depended on weather conditions in Shengsi. Two water sources were generally provided, i.e. reservoir water from 8:00 a.m. to 17:00 p.m., desalinated water from 17:00 p.m. to 8:00 a.m. The amount of desalinated water supply would be increased when rainfall was not enough, and even reach to 100% if no water was stored. More than half of the respondents said they knew the water supply pattern in Shengsi.

#### Safety of desalinated water

In order to evaluate the safety of desalinated water, 12 indicators (including important microbial, physical and chemical indicators) are used in the daily test of desalinated water. Routine tests are held once a month and a complete analysis is conducted every six months according to the requirement of “Standards for Drinking Water Quality” (GB5749). Meanwhile, one respondent also expressed some concern," *We don’t know whether the desalinated water contains other unknown substances except the target substances*, *and if so*, *whether it would harm people*.” However, the medical staffs said that there was no evidence indicating the rise of certain disease morbidity and mortality since the supply of desalinated water in Shengsi Island. Safety concern about desalinated water among residents mainly focused on the degree of corrosion of water supply equipment, "*The water faucet could be used for a longer time before the use of desalinated water*, *while we need to change it within 3 years now*, *and we don’t use rust water first running from the tap*." Both the tests results from Shengsi CDC and waterplants indicated that the acid degree of desalinated water (PH between 6.5–6.7) was higher than fresh water.

### Attitudes to desalinated water

The majority of respondents accepted the desalination and believed that desalination is a good way to mitigate water shortages. However, some still showed concerns. "*It is only used here*. *We don’t want be guinea pigs* “; "*I don’t dare to use it*, *I don’t know why*, *just feel bad*." A doctor said that attention should be paid to long-term accumulative effects, "*It may not be a problem now*, *but would be a problem after a few decades*. *No one can be sure about that*.”

One participant had opposite viewpoint: "*Most of the necessary nutrients our body need come from food rather than water*, *so I don’t care about what was less in desalinated water*. *No one got sick in drinking pure water*, *but more problems were found in people drinking un-treated tap water*. "

All of the respondents agreed that the government should conduct desalinated water safety assessments, and develop desalinated water quality and health standards actively. They believed that the governments should take measures to ensure the safety of desalinated water and help more people to establish confidence.

### Practice of desalinated water usage

The water used by the residents in Shengsi islands mainly comes from three sources, i.e. reservoir water (rainfall), desalinated water and bottled purified water (rainfall).

Half of respondents said whether desalinated water was supplied or not did not affect their choice. Others, who had higher education level, preferred purified water and only used desalinated water for washing. "*I don’t use running water for cooking and drinking after 17*:*00 p*.*m*. *every day*, *we prefer purified water*." A director of one purified water company confirmed that the source of water using in purified-water production was collected rainfall. He said "*My family use tap water all the time*, *regardless of what kind of water*, *I think there is no difference*."

A director of water conservancy department suggested conducting a double pipeline (desalinated water and drinking water for each) project and setting ladder-type price for different water.

## Discussion

Water scarcity is estimated to affect one third of people worldwide and the situation is expected to be worsening due to the increasing need for water along with population growth, urbanization, climate change and more household and industrial uses [1, 6]. China, with the largest population in the world, will be affected seriously too. This situation has made seawater desalination a fast-growing alternative [1]. However, previous studies mainly focused on its environmental impacts, energy consumption and desalination technologies rather than the effect on human health [2, 7–9]. Our study was the first KAP study among desalinated water users.

Shengsi County, located in the far north of Zhoushan islands in Zhejiang Province, is composed of islands and isolated from continent. The local average annual rainfall is about 1000 millimeters; Shengsi County is one of the water-stressed island counties in China and one of the places using desalinated water as drinking water [5, 10]. Thereby, we selected this county as our study site.

With in-depth interview and focus group discussion, we learnt more about the knowledge, attitudes and practice of 17 professionals working in health and water industries to desalinated water. Our study results showed that some people still had misgivings about desalinated water. Although they knew the basic information about desalination, they only used it for washing purpose. According to the staff from the waterworks and conservancy department, although the cost of desalinated water (5 yuan RMB/ton) is higher than those of domestic water (2.4∼4.9 yuan RMB/ton) and industry use water (3.3∼7.85 yuan RMB/ton), the price was still the same for both sources of water for users. Therefore, we indicated that the cost of desalinated water is not the main factor affecting the promotion of desalinated water use, while the attitude and practice are the key factors. All of the respondents believed the government should develop comprehensive quality standards for desalinated water and establish the public’s confidence on desalinated water.

As far as we know, no report has confirmed that desalinated water is harmful to human health. While there still has something we need to pay attention. Previous studies regularly monitored the desalination factory water and tap water, respectively. Data showed that after reverse osmosis, both factory water and tap water met the requirement of “Standards for Drinking Water Quality"(GB5749) [11], except some items, such as turbidity, free residual chlorine and boron. Free residual chlorine indicator failed to reach the standards occasionally due to the improper pretreatment and inaccurate amount of chlorine using in sterilization. In terms of key contaminants directly affecting people’s health, the most important one is probably boron, which might exist at high concentration in seawater and might not be removed completely by reverse osmosis plants. Former studies indicated that the concentration of boron in desalinated water in Shengsi ranged from 0.7 to 1.5mg/L [10, 12], higher than the threshold in GB5749, but lower than the drinking-water standard developed by the World Health Organization (WHO) (2.4 mg/L)[13], so we supposed this concentration of boron may have little impact on human health.

The current drinking water standard doesn’t address desalination as unique issues, however, WHO has developed the Guidelines for Drinking Water Quality (GDWQ)[14], which could apply to both conventional and unconventional drinking water production, including desalination.

One obvious problem of desalinated water found by local people was corrosion, which was consistent with a previous study report [15]. The corrosion level should be reduced to a level that the concentration of dissolved metals used in pipes doesn’t exceed standard drinking water guidelines. Local government had realized this problem and began to improve the water pipe network in Shengsi in 2007, using PV pipes to replace the original iron pipes.

Only one of the respondents mentioned about the mineral nutrition in desalinated water and showed no worry. Considering the fact that the objective of desalination is to reduce or remove all the substances in seawater, including inorganic ions, we need to pay attention to this issue. Calcium and magnesium are essential nutrients and the principal defining components affecting “water hardness”, and also can improve the stability of desalinated water. Although calcium and magnesium are often added to desalinated water, the hardness of desalinated water measured in previous studies was still at a low level with 7∼99mg/L [4, 10, 12]. Many studies suggested that drinking water hardness had a negative correlation with cardiovascular disease mortality [16–18], and also with cancer, diabetes and low birth weight infants [19, 20].Thereby, these ions perhaps need to be considered carefully by setting a low limit. More systematic testing and evaluation system and technical standards are necessary for promoting seawater desalination.

The subjects we recruited in our study were professionals in health and water industries. They were more knowledgeable about desalinated water and had more comprehensive understanding of desalinated water; this would bring some cognitive bias. Compared with the ordinary residents of Shengsi County, these subjects may be more concerned about the health problems, therefore more cautious when making choices in front of potential risks. In addition, some bias was also brought when asking subjects the behavior and attitude of people around them due to recall bias and personal understanding. Therefore, one should be cautious in the use of these results in guiding water desalination research and policies-making, and need to give full consideration to the limitation that mentioned above. Further, we want to have an epidemiological survey in this place to understand more KAP of desalination among ordinary citizens.

Drinking water systems should strive to produce and deliver safe drinking water that meets all health quality specifications. People (even those with medical backgrounds and working in the water industry) are voicing some concerns about the water quality and therefore quality standards may help to allay those concerns. At the same time, more comprehensive recognition of the great societal and ecological benefits of using seawater resources—economic vitality, public health, national security, and ecosystem health—should further motivate the search for technological solutions to water shortages. Among key issues constraining the development of desalination, new technologies, new processes and new products with independent intellectual property rights, as well as new membrane and membrane material, equipment and other core technologies, are the most important ones at present time. To promote the use of desalinated water and desalination industry in China, we also need to not only increase awareness of saving water, but also make people fully aware of the feasibility and safety of seawater desalination.

## References

[1] Service RF. Desalination freshens up. Science. 2006; 313(5790): 1088–1090. 10.1126/science.313.5790.1088 16931754

[2] Elimelech M, Phillip WA. The future of seawater desalination: energy, technology, and the environment. Science. 2011; 333(6043): 712–717. 10.1126/science.1200488 21817042

[3] Ministry of Science and Technology of People’s Republic of China & National Development and Reform Commission. The 12th Five-Year Plan for the scientific and technological development of seawater desalinization. 2012. Available: http://www.gov.cn/zwgk/2012-08/29/content_2212933.htm

[4] Zhang Y, Ying N, Zheng W, Fang H, Ni H. Quality research and analysis of desalinated water in Shengsi. Chinese J Health Lab Tech. 2007; 17(2): 371–372.

[5] Ni H, Jiang Z, Fei J, Guo C. Detection of treated water by reverse osmosis method for seawater desalination in Shengsi island from 2007 to 2008. Chinese J Health Lab Tech. 2010; 20(1): 162–164.

[6] WHO. Safe Drinking-water from Desalination. WHO/HSE/WSH/11.03.2011. Available: http://apps.who.int/iris/bitstream/10665/70621/1/WHO_HSE_WSH_11.03_eng.pdf

[7] Peñate B, García-Rodrígue L. Current trends and future prospects in the design of seawater reverse osmosis desalination technology. Desalination.2012; 284: 1–8. 10.1016/j.desal.2011.09.010

[8] Tsiourtis NX. Desalination and the environment. Desalination. 2001; 141(3): 223–236. 10.1016/s0011-9164(01)85001-3

[9] Meerganz von Medeazza GL. "Direct'' and socially-induced environmental impacts of desalination. Desalination. 2005; 185(1): 57–70. 10.1016/j.desal.2005.03.071

[10] Zhang Y, Wang H, Yu X, Tong Z. Surveillance Data Analysis of Multi-Sources of Water and Drinking Water. J Environ Occup Med. 2012; 29(11): 681–688.

[11] Ministry of Health of People’s Republic of China Standard for drinking water quality (GB5749-2006) Beijing: China Standard Press; 2006.

[12] Zhao X, Xu C, Zhang M, Zheng L, Chen X, Hu X, et al Hygienic investigation on desalinated seawater in an island. J Environ Health. 2013; 30(4): 335–338.

[13] WHO. Boron in drinking‐water. Background document for development of WHO Guidelines for Drinking‐water Quality. 2009. Available: http://www.who.int/water_sanitation_health/dwq/wsh0304_54/en/index8.html.

[14] WHO. Guidelines for drinking-water quality The fourth edition WHO; 2011.

[15] Cotruvo J, Voutchkov N, Fawell J, Payment P, Cunliffe D, Lattemann S. Desalination technology: health and environmental impacts Boca Raton, USA: Taylor and Francis Group, CRC Press; 2010.

[16] Kousa A, Moltchanova E, Viik-Kajander M, Rytkonen M, Tuomilehto J, Tarvainen T, et al Geochemistry of ground water and the incidence of acute myocardial infarction in Finland. J Epidemiol Community Health. 2004; 58(2): 136–139. 10.1136/jech.58.2.136 14729895 PMC1732675

[17] Marque S, Jacqmin-Gadda H, Dartigues JF, Commenges D. Cardiovascular mortality and calcium and magnesium in drinking water: an ecological study in elderly people. Eur J Epidemiol. 2003;18(4): 305–309. 10.1023/A:1023618728056 12803370

[18] Nerbrand C, Agreus L, Lenner RA, Nyberg P, Svardsudd K. The influence of calcium and magnesium in drinking water and diet on cardiovascular risk factors in individuals living in hard and soft water areas with differences in cardiovascular mortality. BMC public Health. 2003; 3(1): 21 10.1186/1471-2458-3-21 12814520 PMC198279

[19] Chiu HF, Chang CC, Chen CC, Yang CY. Calcium and magnesium in drinking water and risk of death from kidney cancer. J Toxicol Environ Health A. 2011; 74(1): 62–70. 10.1080/15287394.2010.514236 21120749

[20] Zhao HX, Mold MD, Stenhouse EA, Bird SC, Wright DE, Demaine AG, et al Drinking water composition and childhood-onset Type 1 diabetes mellitus in Devon and Cornwall, England. Diabet Med. 2001;18(9): 709–717. 10.1046/j.1464-5491.2001.00554.x 11606168

